# The efficacy and safety of autologous blood transfusion drainage in patients undergoing total knee arthroplasty: a meta-analysis of 16 randomized controlled trials

**DOI:** 10.1186/s12891-016-1301-7

**Published:** 2016-11-02

**Authors:** Jian-ke Pan, Kun-hao Hong, Hui Xie, Ming-hui Luo, Da Guo, Jun Liu

**Affiliations:** 1 Department of Orthopedics, Second Affiliated Hospital of Guangzhou University of Chinese Medicine (Guangdong Provincial Hospital of Chinese Medicine), No. 111 Dade Road, Guangzhou, Guangdong 510120 China; 2 Department of Orthopedics, Guangdong Second Traditional Chinese Medicine Hospital, No. 60 Hengfu Road, Guangzhou, Guangdong 510095 China

**Keywords:** Autologous blood transfusion drainage, Total knee arthroplasty, Meta-analysis, Randomized controlled trials

## Abstract

**Background:**

Autologous blood transfusion drainage (ABTD) has been used for many years to reduce blood loss in total knee arthroplasty (TKA). We evaluate the current evidence concerning the efficiency and safety of ABTD used in TKA compared with conventional suction drainage (CSD).

**Methods:**

We performed a systematic literature search of the PubMed, Embase, Cochrane Library and four Chinese databases. All randomized controlled trials (RCTs) that compared the effects of ABTD versus CSD in TKA were included in the meta-analysis.

**Results:**

Sixteen RCTs involving 1534 patients who compared the effects of ABTD versus CSD were included. Five of the RCTs were performed in Asia, ten in Europe, and one in North America. Patients in the ABTD group had a lower blood transfusion rate (OR: 0.25 [0.13, 0.47]; *Z* = 4.27, *P* < 0.0001) and fewer units transfused per patient (WMD: −0.68 [−0.98, −0.39]; *Z* = 4. 52, *P* < 0.00001) than did patients in the CSD group. Wound complications, deep vein thrombosis, febrile complications, post-operative hemoglobin days 5–8, drainage volume, and length of hospital stay did not differ significantly between the two types of drainage systems.

**Conclusion:**

This meta-analysis suggests that ABTD is a safe and effective method that yields a lower blood transfusion rate and fewer units transfused per patient in TKA compared with CSD.

**Electronic supplementary material:**

The online version of this article (doi:10.1186/s12891-016-1301-7) contains supplementary material, which is available to authorized users.

## Background

Total knee arthroplasty (TKA) can result in significant blood loss [1, 2]. One study estimated the average blood loos in TKA to be 1500 mL [3]. The average reduction in Hb concentration after TKA has been estimated to be 3.85 g/dL [4]. Blood transfusion may be considered necessary in some patients to avoid symptomatic anemia and subsequent delays in postoperative rehabilitation [5]. The blood transfusion rate in TKA reach 39 % [2, 6, 7].

Conventional suction drainage (CSD) is used worldwide for postoperative wound blood collection in TKA [8]. Formerly, CSD was believed to be effective in decreasing hematoma formation [9–11] and potentially able to decrease postoperative pain, swelling, and the incidence of infection [12]. However, a closed suction drainage system increases bleeding because it eliminates the tamponade effect of a closed, undrained wound [8]. Surgeons use adjunctive measures such as autologous blood transfusion drainage (ABTD) to reduce excessive blood loss from the drain [13], and recent studies have shown that ABTD can decrease the rate of blood transfusion [14–16].

The efficacy and safety of ABTD in the management of a patient’s blood during TKA surgery was assessed in a previous meta-analysis of eight randomized controlled trials (RCTs) [17]. The meta-analysis showed that ABTD was superior to CSD with respect to the blood transfusion rate (OR: 0.25 [0.13, 0.47]; *P* < 0.0001), the number of units transfused per patient (WMD: −0.84 [−1.13, −0.56]; *P* < 0.0001), and the length of hospital stay (WMD: −0.25 [−0.48, −0.01]; *P* = 0.04). However, data extraction errors involving the number of patients requiring homologous blood transfusion occurred with two of the studies [18, 19] included in the meta-analysis. Furthermore, the meta-analysis did not employ intention-to-treat (ITT) analysis, which may have led to anti-conservative estimates of treatment effectiveness. In addition, systematic reviews that fail to search non-English databases may miss relevant studies and cause selection bias [20]. As trials with statistically significant results are more likely to be published in English than are those with non-significant results [21], systematic reviews that include studies published only in English might overestimate true effects, In addition, the previous meta-analysis did not evaluate the outcomes of additional measures such as wound complications (including wound infection, wound abscess, wound dehiscence, and wound hematoma) and febrile complications.

In recent years, several studies comparing ABTD and CSD have reported conflicting outcomes [1, 22–24]. Whether the benefits of ABTD are limited to the reduction of the blood transfusion rate is unclear. Therefore, we comprehensively searched several bibliographic databases to identify RCTs conducted to date. We then analyzed the clinical evidence to evaluate the effectiveness and safety of ABTD relative to CSD. We also investigated the potential benefits of ABTD.

## Methods

In accordance with Preferred Reporting Items for Systematic Reviews and Meta-analysis [25], we made a prospective protocol of objectives, literature-search strategies, inclusion and exclusion criteria, outcome measurements, and methods of statistical analysis before the research began.

### Data sources and search strategies

A systematic literature search of the Pubmed (1950–February 2016), Embase (1974–February 2016), Cochrane Library (February 2016 Issue 2), Chinese Biomedical Literature (CBM) (1990 to February 2016), China National Knowledge Infrastructure (CNKI) (1979 to February 2016), Chinese Scientific Journals (VIP) (1989 to February 2016) and Wanfang (1982 to February 2016) databases was conducted. The following MeSH terms or Emtree terms and their combinations were searched in [Title/Abstract]: “Drainage”, “Suction”, “Blood Transfusion, Autologous”, “Operative Blood Salvage”, “Arthroplasty, Replacement, Knee” or “wound drainage”, “closed drainage”, “drainage catheter”, “drainage tube”, “suction drain”, “surgical drainage”, “drain”, “wound drain”, “blood autotransfusion”, “autotransfusion unit”, “blood salvage”, “knee arthroplasty”. (See Additional file 1 for details on the search strategies.) Only articles that were originally written in English or Chinese or that had been translated into English were considered. Unpublished trials were not included. When multiple reports describing the same population were published, the most recent or complete report was used. Additional eligible studies were sought by searching the reference lists of primary articles and relevant reviews.

### Inclusion criteria

All available RCTs that compared ABTD with CSD in TKA and for which one or more comparable quantitative outcomes (the quantitative data must be presented as means and standard deviations or 95 % confidence intervals) could be extracted and analyzed were included.

### Exclusion criteria

We excluded case reports, non-original research (e.g., review articles, editorials, letters to the editor), non-human animal studies, and duplicate publications.

### Data extraction and analysis

Data abstraction was conducted by two authors (Hong and Xie) independently. In cases of disagreement, consensus was established through discussion with two other experienced authors (Pan and J. Liu).

The primary outcomes were blood transfusion rate, mean number of units transfused per patient, wound complications, and deep vein thrombosis.

The secondary outcomes were febrile complications, post-operative hemoglobin on days 5–8, drainage volume, and length of hospital stay.

### Quality assessment

The Jadad quality scale [26] and the Cochrane risk of bias tool [27] were used to assess the methodological quality of the included RCTs. Studies with a Jadad score ≥ 3 were considered high quality, and those with a Jadad score ≤2 were considered low quality.

### Data synthesis and analysis

We based our analysis on intent-to-treat (ITT) or modified ITT data. Review Manager 5.3.5 (Cochrane) was employed for the meta-analysis. Odds ratios (ORs) and 95 % confidence intervals (CIs) were calculated for blood transfusion rate, wound complications, deep vein thrombosis and febrile complications. Weighted mean differences (WMDs) and 95 % CIs were calculated for the mean number of units transfused per patient, post-operative hemoglobin on days 5–8, drainage volume, and length of hospital stay. We regarded the volume of one unit of transfused blood as approximately 300 mL [18, 28]. When continuous data from the included studies were presented as means and 95 % confidence intervals, standard deviations were calculated by using Review Manager 5.3.5 (Cochrane).

Heterogeneity among the studies was assessed using the I-square test. Where heterogeneity (*I* ^2^ > 50 %) was detected, a random-effects model was applied; otherwise, a fixed-effects model was applied [27]. For outcome measures with I^2^ values greater than 50 %, we conducted sensitivity analyses to determine the source. Funnel plots were inspected visually to assess the possibility of publication bias.

## Results

### Study selection

**Fig. 1:**
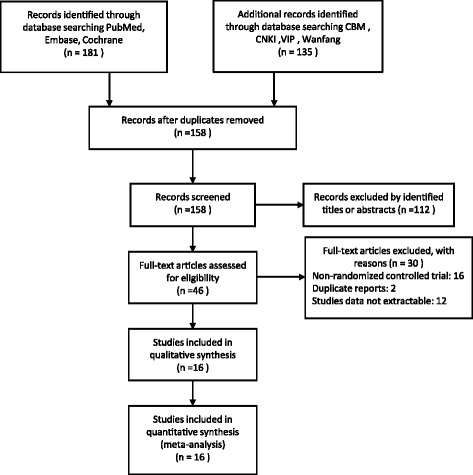
Flow diagram of studies identified, included, and excluded

Sixteen [18, 19, 29–42] studies including 1534 cases (769 cases for ABTD and 765 cases for CSD) met the inclusion criteria and were included in the final analysis (Fig. 1). Search of the reference lists revealed no additional studies that met the inclusion criteria.

### Characteristics of the included studies

The characteristics of the included studies are summarized in Table 1. Four studies [39–42] were identified from Chinese databases, and 12 studies [18, 19, 29–38] were identified from international databases. Geographically, five RCTs were performed in Asia, 10 in Europe, and one in North America.

**Table 1:** Characteristics of the included studies

Study	Country	Jadad score	Patients, no.		Surgical method	Age^a^		M:F ratio		Pre-op Hb^a^	
			ABTD	CSD		ABTD	CSD	ABTD	CSD	ABTD	CSD
Deng YJ 2015 [40]	China	1	11	12	B-TKA	57.7 ± 16.3	60.7 ± 17.3	5:6	5:7	13.4 ± 3.6	13.5 ± 3.7
Jin CH 2014 [42]	China	1	70	70	SU-TKA	66 ± 4	64 ± 4	12:58	13:57	13.1 ± 1.3	13. 2 ± 1. 4
Sun YT 2014 [39]	China	1	72	60	SU-TKA	65.3	64.7	15:57	13:47	13.1 ± 1.4	13.4 ± 2.0
Amin A 2008 [29]	UK	2	92	86	SU-TKA	70.3	70.4	43:49	39:47	13.2 ± 1.2	13.4 ± 1.3
Shen Y 2007 [41]	China	0	60	60	SU-TKA	NA	NA	NA	NA	NA	NA
Zacharopoulos A 2007 [30]	Greece	1	30	30	SU-TKA	69.2	70.2	6:24	7:23	NA	NA
Abuzakuk T 2007 [31]	UK	2	52	52	SU-TKA	NA	NA	21:31	22:30	13.6 ± 1.5	13.5 ± 1.2
Kirkos JM 2006 [32]	Greece	0	78	77	SU-TKA	69.1 ± 5.5	68.9 ± 5.1	18:60	10:67	13.0 ± 1.4	13.1 ± 1.4
Dramis A 2006 [33]	UK	1	25	24	SU-TKA	NA	NA	NA	NA	NA	NA
Cheng SC 2005 [34]	China	2	26	34	SU-TKA	72	69.6	6:20	12:22	12.4	12.8
Thomas D 2001 [35]	UK	2	115	116	SU-TKA	NA	NA	44:71	55:61	NA	NA
Breakwell LM 2000 [36]	UK	1	14	19	B-TKA	66.8	73.7	8:6	8:11	12.9	12.8
Adalberth G 1998 [18]	Sweden	3	30	30	SU-TKA	71 ± 5.4	72 ± 8.0	NA	NA	13.8 ± 1.1	14.3 ± 1.3
Newman J 1997 [37]	UK	2	35	35	SU-TKA	NA	NA	NA	NA	13.4 ± 1.2	13.2 ± 1.4
Heddle NM 1992 [19]	Canada	3	39	40	SU-TKA	69.3 ± 6.9	71.0 ± 9.0	25:14	26:14	NA	NA
Majkowski RS 1991 [38]	UK	1	20	20	SU-TKA	71.3	70.3	6:14	6:14	13.2	12.7

*SU-TKA* selective unilateral total knee replacement, *B-TKA* bilateral total knee replacement, *ABTD* autologous blood transfusion drainage, *CSD* conventional suction drain, *NA* data not available, *M* male, *F* female, *Pre-op Hb* pre-operative hemoglobin

^a^Mean or Mean ± SD

**Fig. 2:**
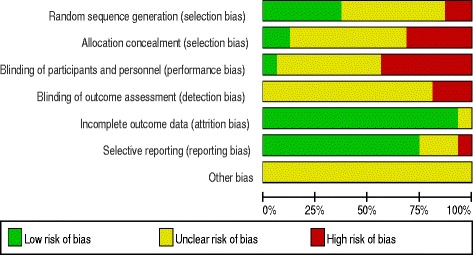
Risk of bias summary

**Fig. 3:**
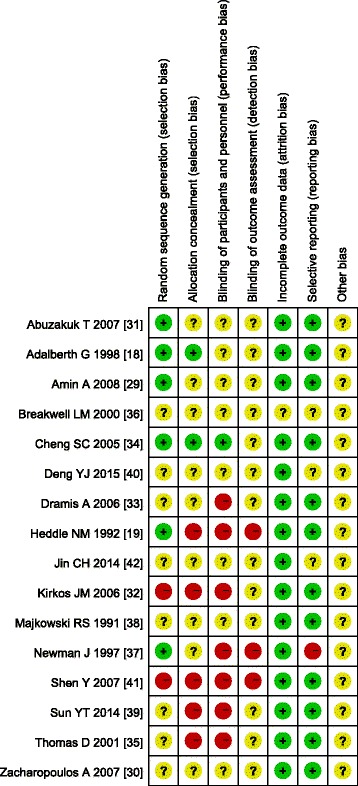
Risk of bias assessment

We evaluated the methodological quality of all of the included studies using the Jadad quality scale and Cochrane risk of bias criteria (Table 1, Figs. 2 and 3). The Jadad scores ranged from 0 to 3 points, with an average score of 1.4. Only two RCTs [18, 19] were of high quality. Six studies [18, 19, 29, 31, 34, 37] reported a method of randomization, and two studies [32, 41] used a method of quasi-randomization. The remaining eight studies [30, 33, 35, 36, 38–40, 42] did not report the method of randomization. None of the included studies used the double-blinded method. The two RCTs [18, 19] of high quality described the number of cases and the reasons for drop-out in detail. Two studies [18, 34] reported the method of allocation concealment. One study [34] provided information regarding the blinding method. None of the 16 studies [18, 19, 29–42] reported the method of blinding outcome assessment. Fifteen studies [18, 19, 29–35, 37–42] reported the complete analysis. One study [37] was at high risk of selective reporting.

Patients in 14 studies [18, 19, 29–35, 37–39, 41, 42] were undergoing selective unilateral TKA, and those of the remaining two [36, 40] were undergoing bilateral TKA.

The majority of the RCTs reviewed in this meta-analysis were of low quality. All of the included studies reported that the baseline characteristics of the study groups, including age, gender and pre-operative hemoglobin, were comparable, as shown in Table 1.

### Primary outcomes

#### Blood transfusion rate

**Fig. 4:**
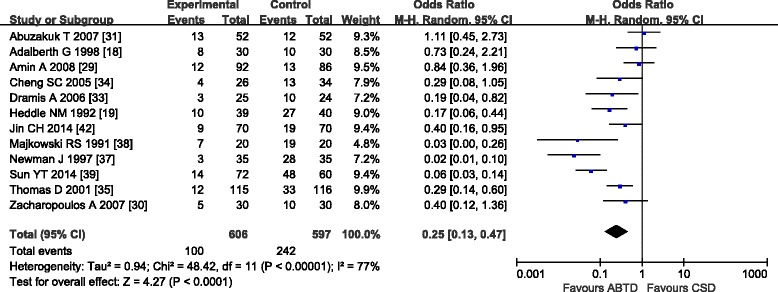
Blood transfusion rate

Twelve trials [18, 19, 29–31, 33–35, 37–39, 42] compared ABTD with CSD in the number of patients requiring homologous blood transfusion. Ten trials [18, 19, 29–31, 33–35, 37–39, 42] showed substantial heterogeneity in the trial results (chi-square = 48.42, *P* < 0.00001; *I* ^2^ = 77 %). Therefore, a random effects model was used for statistical analysis. The meta-analysis showed a significant beneficial effect of ABTD compared with CSD on blood transfusion rate (16.50 and 40.54 %, respectively; OR: 0.25 [0.13, 0.47]; *Z* = 4.27, *P* < 0.0001) (Fig. 4). Due to marked heterogeneity in the blood transfusion rate data, sensitivity analysis was conducted by excluding one study randomly. Dropping any one study did not reduce the heterogeneity, suggesting that the result was robust against the heterogeneity.

#### Mean number of units transfused per patient

**Fig. 5:**
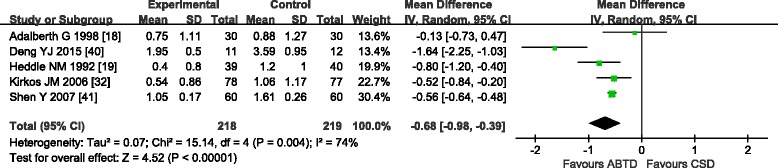
Mean number of units transfused per patient

Five trials [18, 19, 32, 40, 41] that included a total of 437 patients reported the mean number of units transfused per patient (Fig. 5). These five trials [18, 19, 32, 40, 41] showed moderate heterogeneity in the results (chi-square = 15.14, *P* = 0.004; *I* ^2^ = 74 %). Therefore, a random effects model was used for statistical analysis. The meta-analysis showed a significant beneficial effect of ABTD compared with CSD; i.e., a lower mean number of units transfused per patient (WMD: −0.68 [−0.98, −0.39]; *Z* = 4.52, *P* < 0.00001). Due to marked heterogeneity in blood transfusion rate, sensitivity analysis was conducted by excluding one study [40] of lower quality, which reduced the heterogeneity (*I* ^2^ = 12 %, *P* = 0.33). The random effects model also showed a significant beneficial effect of ABTD relative to CSD (WMD: −0.56 [−0.68, −0.44]; *Z* = 9.39, *P* < 0.00001). Dropping any one study did not influence the qualitative result.

#### Wound complications

**Fig. 6:**
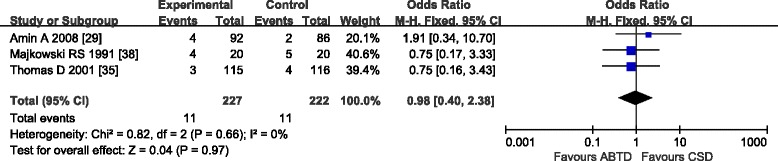
Wound complication

The analysis of data extracted from three studies [29, 35, 38] that assessed wound complications in 449 patients revealed no significant difference between the ABTD and CSD groups (4.85 and 4.95 %, OR: 0.98 [0.40, 2.38]; *Z* = 0.04, *P* = 0.97). No significant heterogeneity was detected (*P* = 0.66, *I* ^2^ = 0 %) (Fig. 6).

#### Deep vein thrombosis

**Fig. 7:**
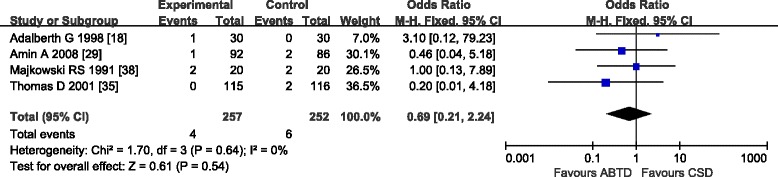
Deep vein thrombosis

Data extracted from four studies [18, 29, 35, 38] that assessed deep vein thrombosis in 509 patients showed no significant difference between the ABTD and CSD groups (1.56 and 2.38 %, OR: 0.69 [0.21, 2.24]; *Z* = 0.61, *P* = 0.54). No significant heterogeneity was detected (*P* = 0.64, *I* ^2^ = 0 %) (Fig. 7).

### Secondary outcomes

#### Febrile complications

**Fig. 8:**
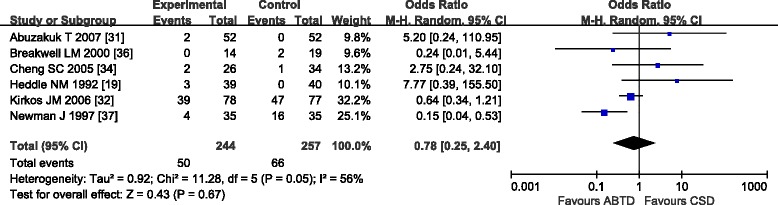
Febrile complications

Six trials [19, 31, 32, 34, 36, 37] compared ABTD with CSD with respect to febrile complications. These six trials showed substantial heterogeneity in the results (chi-square = 11.28, *P* = 0.05; *I* ^2^ = 56 %); therefore, a random effects model was used. The meta-analysis showed no significant difference between the two groups (20.49 and 25.68 %, OR: 0.78 [0.25, 2.40]; *Z* = 0.43, *P* = 0.67) (Fig. 8). Due to marked heterogeneity in the febrile complications data, sensitivity analysis was conducted by excluding one study [37] of lower quality, which reduced the heterogeneity (*I* ^2^ = 30 %, *P* = 0.22). The random effects model also showed no significant difference between the ABTD and CSD groups (21.01 and 22.52 %, OR: 1.21 [0.39, 3.68]; *Z* = 0.33, *P* = 0.74). Dropping any one study did not qualitatively alter the result.

#### Post-operative hemoglobin on days 5–8

**Fig. 9:**
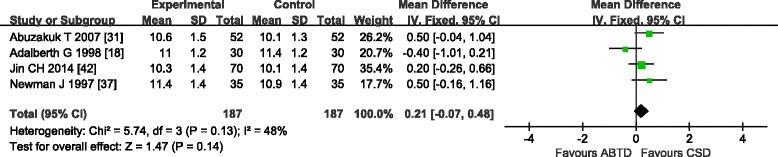
Post-operative haemoglobin days 5–8

Four studies [18, 31, 37, 42] reported post-operative hemoglobin on days 5–8. Among these studies, one [31] reported hemoglobin on the fifth day post-operation, one [18] reported hemoglobin on the eighth day post-operation, and the remaining two [37, 42] reported hemoglobin on the seventh day post-operation. Because the four studies [18, 31, 37, 42] showed moderate heterogeneity in the results (chi-square = 5.74, *P* = 0.13; *I* ^2^ = 48 %), a fixed effects model was used. The meta-analysis showed a significant beneficial effect of CSD compared with ABTD on post-operative hemoglobin on days 5–8 (WMD: 0.21 [−0.07, 0.48]; Z = 1.47, *P* = 0.14) (Fig. 9).

#### Drainage volume

**Fig. 10:**
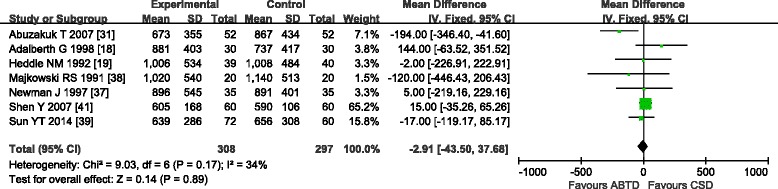
Drainage volume

Seven studies [18, 19, 31, 37–39, 41] reported post-operative drainage volume. These seven studies showed moderate heterogeneity in the results (chi-square = 9.03, *P* = 0.17; *I* ^2^ = 34 %); therefore, a fixed effects model was used. Pooling and analysis of the data of the 605 patients from the seven studies revealed no significant difference between the ABTD and CSD groups (WMD: −2.91 [−43.50, 37.68]; Z =0.14, *P* = 0.89) (Fig. 10).

#### Length of hospital stay

**Fig. 11:**
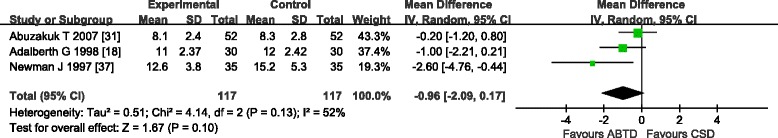
Length of hospital stay

Three trials [18, 31, 37] compared ABTD with CSD in length of hospital stay. The three trials [18, 31, 37] showed substantial heterogeneity in the results (chi-square = 4.14, *P* = 0.13; *I* ^2^ = 52 %); therefore, a random effects model was used. The meta-analysis showed no significant difference in length of hospital stay between the ABTD and CSD groups (WMD: −0.96 [−2.09, 0.17]; *Z* = 1.67, *P* = 0.10) (Fig. 11). Due to marked heterogeneity in length of hospital stay, sensitivity analysis was conducted by excluding one study [37] of lower quality, resulting in no significant heterogeneity detected (*P* = 0.32, *I* ^2^ = 0 %). The random effects model also showed no significant difference between the two groups in length of hospital stay (WMD: −0.52 [−1.30, 0.25]; *Z* = 1.33, *P* = 0.18).

#### Publication bias

**Fig. 12:**
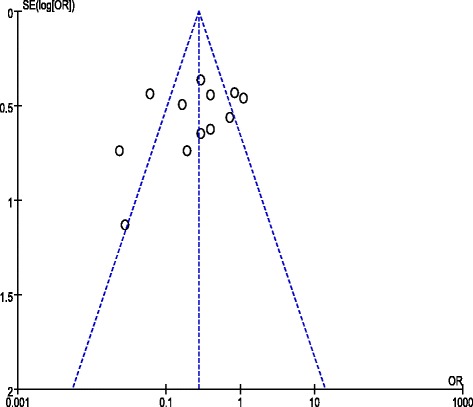
Funnel plot of blood transfusion rate

The funnel plot of blood transfusion rate (Fig. 12) showed a markedly asymmetrical distribution of effect estimate, which indicated the presence of publication bias.

## Discussion

The meta-analysis of 16 RCTs, including 1534 patients, suggested that ABTD is a safe system that yields a significantly reduced blood transfusion rate and fewer units of transfused blood per patient compared with CSD. We found no significant differences between the two drainage systems in wound complications, deep vein thrombosis, febrile complications, post-operative hemoglobin on days 5–8, drainage volume, or length of hospital stay.

TKA patients require post-operative allogenic blood transfusion, which was markedly reduced by using ABTD compared with CSD. Although allogenic transfusion remains the most popular method of compensating for blood loss in TKA patients, it can have potential deleterious effects, including transfusion-related infection, incompatibility-related transfusion reaction, immune modulatory effects, and febrile complications [34]. These risks have led to use of autologous pre-donation blood, which also has drawbacks, e.g., difficulty of organizing patients for pre-donation and adherence to iron or erythropoietin therapy [43]. Studies have reported that nearly half of the autologous blood donated by patients for surgery is discarded [44, 45]. The use of autologous pre-donation blood is wasteful and costly [46]. Compared with the use of autologous pre-donation blood, ABTD has been found to be easier to perform, more cost-effective and able to lower the risks associated with allogenic blood use [47]. The present meta-analysis found that ABTD showed a significantly reduced blood transfusion rate and number of units transfused per patient; therefore, although the ABTD device is more expensive than CSD, a TKA patient using ABTD could spend 20 to 70 % less money on allogenic blood transfusion [30, 32, 34–36]. The procedures for setting up an ABTD system are similar to those for standard allogeneic blood transfusions [34] and require no additional medical personnel, but they do add staff time [31, 35]. The exact costs saved by using ABTD was not quantified in the present study because the unit cost of allogenic blood varies among regions.

Analysis of the extracted data on postoperative outcomes demonstrated that ABTD is safe and effective for TKA. There were no significant differences between ABTD and CSD in wound complications, deep vein thrombosis, and febrile complications. Kristiansson et al. [48] found that hypercoagulability and high concentrations of IL-6 were present in drained blood. Some studies have reported that drained blood shows decreased platelet counts, pH levels, and clotting factor levels as well as increased fibrin degradation products [49, 50]. Hand et al. [51] identified low levels of methyl methacrylate monomers in filtered blood. Contra-indications to the use of unwashed shed blood have been formulated by the American Association of Blood Banks [52], who suggested that various cytokines are activated in drained blood and may be problematic for some patients if they increased to higher levels more than 6 h after bleeding [53]. In all of the studies included in the present meta-analysis, re-infusion was completed within 6 h post-operation. A lower rate of allogenic blood transfusion may help prevent febrile complications. Postoperative febrile complications were generally observed in the context of major orthopedic surgery, and it has been suggested that the rise in temperature is a response to the surgical procedure [54]. Some previous studies have also reported no difference between ABTD and CSD in the development febrile complications [55–57]. The absence of significant differences in wound complications, deep vein thrombosis, and febrile complications between ABTD and CSD indicate that ABTD is as safe as CSD.

Analysis of the pooled data revealed no significant difference in drainage volume, suggesting that ABTD is equally safe as CSD with respect to wound bleeding.

We found no significant differences between the two systems in post-operative hemoglobin on days 5–8. ABTD was found to be effective in reducing allogeneic blood transfusions but not in achieving high postoperative hemoglobin levels. A high postoperative hemoglobin level has been reported to be associated with better rehabilitation outcomes after TKA [58]. The present findings suggest that ABTD was not useful in achieving high postoperative hemoglobin levels to enhance rehabilitation, similar to the findings of other studies [57, 59].

The present meta-analysis revealed no significant difference in length of hospital stay between ABTD and CSD. However, the previous meta-analysis [17] found a longer length of hospital stay in the CSD group. The data on length of hospital stay in Amin A et al. [29] were presented as means and ranges, and we were unable to obtain the original data by contacting the corresponding author. Therefore, we excluded these data [29], which were included in the previous meta-analysis [17]. Due to country and regional variation in medical insurance policies and social support facilitating discharge, the length of hospital stay could not be used in the present study as a measure of cost [36].

Two studies [36, 40] on bilateral TKA reported different outcomes that could not be synthesized. Due to this limited number of studies and insufficient description of the study methods, the outcomes could not be analyzed in a subgroup analysis.

To assess the impact of one study on the effect estimates, we performed sensitivity analysis by excluding one study with a high weight or of lower quality. The results regarding blood transfusion rate, febrile complications, and length of hospital stay were qualitatively unchanged by this analysis. However, as a result of the sensitivity analysis, the original result regarding post-operative hemoglobin on days 5–8 was changed to favor CSD, and the heterogeneity decreased from 48 to 0 % when the study by Adalberth et al. [18] was excluded. Analysis of the four studies [18, 31, 37, 42] reporting on pre-operative hemoglobin revealed no significant difference between the two systems (Heterogeneity: chi-square = 3.09, *P* = 0.38; *I* ^2^ = 3 %; WMD: −0.07 [−0.33, 0.20]; *Z* = 0.49, *P* = 0.62). Some non-RCT studies [43, 60] have similarly found no significant difference in hemoglobin levels before and after TKA between the ABTD and CSD groups. Another study [31] found that ABTD could prevent a rapid decrease in hemoglobin level during the early post-operative period, although this benefit was no longer present by post-operative day 5. Because only three studies [18, 31, 37] included in this meta-analysis reported on post-operative hemoglobin, further studies are needed to evaluate the benefit of ABTD with respect to post-operative hemoglobin.

### Limitations

This meta-analysis has limitations. We used the Jadad quality scale [26] and the Cochrane risk of bias tool [27] to assess the methodological quality of the included RCTs. According to the Jadad quality scale, the average score of the included studies was 1.4, and only two RCTs [18, 19] were of high quality. The Jadad score places more emphasis on reporting rather than performance, and its advantage is its simplicity and easy implementation. The Jadad score was used in this study because it makes it easy for readers to comprehend the quality of the included studies. Although a lack of adequate allocation concealment have been found in Jadad scores, this domain was assessed using criteria adopted from the Cochrane Handbook. The more important evaluation method used in this study is the Cochrane risk of bias tool.

The majority of the included RCTs were of moderate quality, and their sample sizes were comparatively small. In addition, it appears that the lack of random sequence generation increased the risk of bias.

Another limitation is that adequate information concerning each final outcome was not consistently provided among the 16 studies included. Furthermore, a prerequisite for the initial meta-analysis was the assumption of similarity between the two kinds of surgeries (SU-TKA and B-TKA), which enabled them to be evaluated together.

Fortunately, the results of the sensitivity analyses were similar to those of the original analyses. Another limitation of the present study is the heterogeneity among the included studies, which may reflect inter-study differences in analysis methodology, surgical method (SU-TKA vs. B-TKA), country and racial type. Future systematic reviews should assess different surgical methods, countries and racial types individually when sufficient high-quality RCT data become available. Surgeon experience in various TKA surgical approaches might also influence the results. Finally, as few of the included studies involving long-term follow-up, long-term outcomes could not be evaluated and require further study.

## Conclusions

The present meta-analysis indicated that ABTD is more efficacious than CSD in reducing the blood transfusion rate and the number of units transfused per patient in TKA patients. The two types of drains appear to be equivalent in terms of wound complications, deep vein thrombosis, febrile complications, post-operative hemoglobin on days 5–8, drainage volume, and length of hospital stay. The results of this meta-analysis can help TKA surgeons make clinical decisions. The development of large-volume, well-designed RCTs and clinical trials with extensive follow-up will clarify the advantages and disadvantages of ABTD.

## Additional file

Additional file 1: Search strategies. (DOCX 18 kb)

## Abbreviations

ABTD: Autologous blood transfusion drainage
B-TKA: Bilateral total knee replacement
CBM: Chinese Biomedical Literature
CIs: Confidence intervals
CNKI: China National Knowledge Infrastructure
CSD: Conventional suction drainage
ITT: Intent-to-trea
ORs: Odds ratios
SU-TKA: Selective unilateral total knee replacement
TKA: Total knee arthroplasty
WMDs: Weighted mean differences
