# Supplementary material for: The efficacy and safety of autologous blood transfusion drainage in patients undergoing total knee arthroplasty: a meta-analysis of 16 randomized controlled trials
Source: BMC Musculoskelet Disord. 2016 Nov 2;17:452. doi: 10.1186/s12891-016-1301-7 (PMC5094026; doi:10.1186/s12891-016-1301-7)
Supplement: Additional file 1: — Search strategies. (DOCX 18 kb) [file 12891_2016_1301_MOESM1_ESM.docx]

**Additional files 1: Search strategies**

**Searched in Pubmed**

| Pubmed | 1950–February 2016 | **#1** | **Search (((((((((Drainage[MeSH Terms]) OR Drainage[tiab]) OR Negative-Pressure Wound Therapy[tiab]) OR Suction[MeSH Terms]) OR suction[tiab]) OR Drainage, Suction[tiab]) OR Suction Drainage[tiab]) OR Suction Drainages[tiab]) OR Drain[tiab])** | [**112297**](http://www.ncbi.nlm.nih.gov/pubmed/?cmd=HistorySearch&querykey=5) |
| --- | --- | --- | --- | --- |
|  |  | **#2** | **Search (((((((((((((((((((((((((Blood Transfusion, Autologous[MeSH Terms]) OR operative blood salvage[MeSH Terms]) OR Transfusions, Autologous Blood[Title/Abstract]) OR Blood Transfusions, Autologous[Title/Abstract]) OR Autologous Blood Transfusions[Title/Abstract]) OR Autotransfusion[Title/Abstract]) OR Autotransfusions[Title/Abstract]) OR Transfusion, Autologous Blood[Title/Abstract]) OR Autologous Blood Transfusion[Title/Abstract]) OR Blood Salvage, Operative[Title/Abstract]) OR Salvage, Operative Blood[Title/Abstract]) OR Intraoperative Blood Salvage[Title/Abstract]) OR Blood Salvage, Intraoperative[Title/Abstract]) OR Salvage, Intraoperative Blood[Title/Abstract]) OR Postoperative Blood Salvage[Title/Abstract]) OR Blood Salvage, Postoperative[Title/Abstract]) OR Salvage, Postoperative Blood[Title/Abstract]) OR Post-Operative Blood Salvage[Title/Abstract]) OR Blood Salvage, Post-Operative[Title/Abstract]) OR Post Operative Blood Salvage[Title/Abstract]) OR Salvage, Post-Operative Blood[Title/Abstract]) OR Intraoperative Blood Cell Salvage[Title/Abstract]) OR Blood Transfusion, Autologous[Title/Abstract]) OR operative blood salvage[Title/Abstract]) OR ABT[Title/Abstract]) OR PAT[Title/Abstract]** | [**12657**](http://www.ncbi.nlm.nih.gov/pubmed/?cmd=HistorySearch&querykey=9) |
|  |  | **#3** | **Search (((((((((((((((((((((((((((((((arthroplasty, replacement, knee[MeSH Terms]) OR Arthroplasties, Replacement, Knee[Title/Abstract]) OR Arthroplasty, Knee Replacement[Title/Abstract]) OR Knee Arthroplasty[Title/Abstract]) OR Arthroplasty, Knee[Title/Abstract]) OR Knee Replacement Arthroplasty[Title/Abstract]) OR Replacement Arthroplasties, Knee[Title/Abstract]) OR Replacement Arthroplasty, Knee[Title/Abstract]) OR Arthroplasties, Knee Replacement[Title/Abstract]) OR Knee Replacement Arthroplasties[Title/Abstract]) OR Arthroplasty, Replacement, Partial Knee[Title/Abstract]) OR Partial Knee Replacement[Title/Abstract]) OR Knee Replacement, Partial[Title/Abstract]) OR Unicondylar Knee Replacement[Title/Abstract]) OR Knee Replacement, Unicondylar[Title/Abstract]) OR Unicompartmental Knee Replacement[Title/Abstract]) OR Knee Replacement, Unicompartmental[Title/Abstract]) OR Unicondylar Knee Arthroplasty[Title/Abstract]) OR Arthroplasty, Unicondylar Knee[Title/Abstract]) OR Knee Arthroplasty, Unicondylar[Title/Abstract]) OR Partial Knee Arthroplasty[Title/Abstract]) OR Arthroplasty, Partial Knee[Title/Abstract]) OR Knee Arthroplasty, Partial[Title/Abstract]) OR Unicompartmental Knee Arthroplasty[Title/Abstract]) OR Arthroplasty, Unicompartmental Knee[Title/Abstract]) OR Knee Arthroplasty, Unicompartmental[Title/Abstract]) OR Knee Arthroplasty, Total[Title/Abstract]) OR Replacement, Total Knee[Title/Abstract]) OR Total Knee Replacement[Title/Abstract]) OR Knee Replacement, Total[Title/Abstract]) OR TKA[Title/Abstract]) OR TKR[Title/Abstract]** | [**19923**](http://www.ncbi.nlm.nih.gov/pubmed/?cmd=HistorySearch&querykey=11) |
|  |  | **#4** | **#1 and #2 and #3** | **113** |
|  |  | **#5** | **Search (((((randomized controlled trial [pt]) OR controlled clinical trial [pt]) OR randomized [tiab]) OR randomly [tiab]) OR trial [tiab]) OR groups [tiab]** | [**2123092**](http://www.ncbi.nlm.nih.gov/pubmed/?cmd=HistorySearch&querykey=13) |
|  |  | **#6** | **Search (animals [mh] NOT humans [mh])** | [**3981705**](http://www.ncbi.nlm.nih.gov/pubmed/?cmd=HistorySearch&querykey=14) |
|  |  | **#7** | **Search (#5 NOT #6)** | [**1778846**](http://www.ncbi.nlm.nih.gov/pubmed/?cmd=HistorySearch&querykey=15) |
|  |  | **#8** | **#4 and #7** | **55** |

**Searched in Embase**

| Embase | 1974–February 2016 | **#1** | **'wound drainage'/exp OR 'wound drainage' OR 'closed drainage'/exp OR 'closed drainage' OR 'drainage catheter'/exp OR 'drainage catheter' OR 'drainage tube'/exp OR 'drainage tube' OR 'surgical drainage'/exp OR 'surgical drainage' OR 'drain'/exp OR 'drain' OR 'suction drain'/exp OR 'suction drain' OR 'drainage' OR 'negative-pressure wound therapy'/exp OR 'negative-pressure wound therapy' OR 'suction'/exp OR 'suction' OR 'drainage, suction'/exp OR 'drainage, suction' OR 'suction drainage'/exp OR 'suction drainage' OR 'suction drainages'** | [**164,681**](http://www.embase.com.proxy2.library.jhu.edu/) |
| --- | --- | --- | --- | --- |
|  |  | **#2** | **'blood autotransfusion'/exp OR 'blood autotransfusion' OR 'blood salvage'/exp OR 'blood salvage' OR 'blood transfusion, autologous' OR 'operative blood salvage' OR 'transfusions, autologous blood' OR 'blood transfusions, autologous' OR 'autologous blood transfusions' OR 'autotransfusion' OR 'autotransfusions' OR 'transfusion, autologous blood' OR 'autologous blood transfusion' OR 'blood salvage, operative' OR 'salvage, operative blood' OR 'intraoperative blood salvage' OR 'blood salvage, intraoperative' OR 'salvage, intraoperative blood' OR 'postoperative blood salvage' OR 'blood salvage, postoperative' OR 'salvage, postoperative blood' OR 'post-operative blood salvage' OR 'blood salvage, post-operative' OR 'post operative blood salvage' OR 'salvage, post-operative blood' OR 'intraoperative blood cell salvage' OR 'abt' OR 'pat' OR 'autotransfusion unit'/exp OR 'autotransfusion unit'** | [**115,908**](http://www.embase.com.proxy2.library.jhu.edu/) |
|  |  | **#3** | **'knee arthroplasty'/exp OR 'knee arthroplasty' OR 'arthroplasty, replacement, knee' OR 'arthroplasty, knee replacement' OR 'arthroplasty, knee' OR 'knee replacement arthroplasty' OR 'replacement arthroplasties, knee' OR 'replacement arthroplasty, knee' OR 'arthroplasties, knee replacement' OR 'knee replacement arthroplasties' OR 'arthroplasty, replacement, partial knee' OR 'partial knee replacement' OR 'knee replacement, partial' OR 'unicondylar knee replacement' OR 'knee replacement, unicondylar' OR 'unicompartmental knee replacement' OR 'knee replacement, unicompartmental' OR 'unicondylar knee arthroplasty' OR 'arthroplasty, unicondylar knee' OR 'knee arthroplasty, unicondylar' OR 'partial knee arthroplasty' OR 'arthroplasty, partial knee' OR 'knee arthroplasty, partial' OR 'unicompartmental knee arthroplasty' OR 'arthroplasty, unicompartmental knee' OR 'knee arthroplasty, unicompartmental' OR 'knee arthroplasty, total' OR 'replacement, total knee' OR 'total knee replacement' OR 'knee replacement, total' OR 'tka' OR 'tkr'** | [**28,788**](http://www.embase.com.proxy2.library.jhu.edu/) |
|  |  | **#4** | **#1 AND #2 AND #3** | **143** |
|  |  | **#5** | **'randomized controlled trial'/exp OR 'randomized controlled trial' OR 'randomized controlled trial':de OR 'controlled clinical trial':de OR 'randomized':ab, ti OR 'randomly':ab, ti OR 'trial':ab, ti OR 'groups':ab, ti** | [**2,728,868**](http://www.embase.com.proxy2.library.jhu.edu/) |
|  |  | **#6** | **#4 and #6** | [**69**](http://www.embase.com.proxy2.library.jhu.edu/) |

**Searched in Cochrane Library**

| Cochrane Library | February 2016 Issue 2 | **#1** | **MeSH descriptor: [Drainage] explode all trees** | **2211** |
| --- | --- | --- | --- | --- |
|  |  | **#2** | **MeSH descriptor: [Suction] explode all trees** | **796** |
|  |  | **#3** | **Drainage or Negative-Pressure Wound Therapy or Suction or Drainage, Suction or Suction Drainage or Suction Drainages or Drain:ti, ab, kw (Word variations have been searched)** | **6710** |
|  |  | **#4** | **#1 OR #2 OR #3** | **6710** |
|  |  | **#5** | **MeSH descriptor: [Blood Transfusion, Autologous] explode all trees** | **624** |
|  |  | **#6** | **MeSH descriptor: [Operative Blood Salvage] explode all trees** | **18** |
|  |  | **#7** | **Blood Transfusion, Autologous or operative blood salvage or Transfusions, Autologous Blood or Blood Transfusions, Autologous or Autologous Blood Transfusions or Autotransfusion or Autotransfusions or Transfusion, Autologous Blood or Autologous Blood Transfusion or Blood Salvage, Operative or Salvage, Operative Blood or Intraoperative Blood Salvage or Blood Salvage, Intraoperative or Salvage, Intraoperative Blood or Postoperative Blood Salvage or Blood Salvage, Postoperative or Salvage, Postoperative Blood or Post-Operative Blood Salvage or Blood Salvage, Post-Operative or Post Operative Blood Salvage or Salvage, Post-Operative Blood or Intraoperative Blood Cell Salvage or ABT or PAT:ti, ab, kw (Word variations have been searched)** | **1575** |
|  |  | **#8** | **#5 OR#6 OR #7** | **1575** |
|  |  | **#9** | **MeSH descriptor: [Arthroplasty, Replacement, Knee] explode all trees** | **1723** |
|  |  | **#10** | **Arthroplasties, Replacement, Knee or Arthroplasty, Knee Replacement or Knee Arthroplasty or Arthroplasty, Knee or Knee Replacement Arthroplasty or Replacement Arthroplasties, Knee or Replacement Arthroplasty, Knee or Arthroplasties, Knee Replacement or Knee Replacement Arthroplasties or Arthroplasty, Replacement, Partial Knee or Partial Knee Replacement or Knee Replacement, Partial or Unicondylar Knee Replacement or Knee Replacement, Unicondylar or Unicompartmental Knee Replacement or Knee Replacement, Unicompartmental or Unicondylar Knee Arthroplasty or Arthroplasty, Unicondylar Knee or Knee Arthroplasty, Unicondylar or Partial Knee Arthroplasty or Arthroplasty, Partial Knee or Knee Arthroplasty, Partial or Unicompartmental Knee Arthroplasty or Arthroplasty, Unicompartmental Knee or Knee Arthroplasty, Unicompartmental or Knee Arthroplasty, Total or Replacement, Total Knee or Total Knee Replacement or Knee Replacement, Total'or TKA or TKR:ti, ab, kw (Word variations have been searched)** | **3266** |
|  |  | **#11** | **#9 OR #10** | **3266** |
|  |  | **#12** | **#4 AND # 8 AND #11** | **57** |
